# Vasculo-Behçet's disease with a giant pseudoaneurysm of superficial femoral artery: A case report

**DOI:** 10.1016/j.ijscr.2023.108534

**Published:** 2023-07-24

**Authors:** Mohammad A. Al-doud, Anas N. Al-Nusairat, Nael M. Al-shanableh, Sadeq M. Da'meh, Omar I. Thabcem, Moath M. El-Sageer

**Affiliations:** a Department of Vascular Surgery, Jordanian Royal Medical Services (JRMS), Amman, Jordan; b Department of Anesthesiology and Intensive Care, Jordanian Royal Medical Services (JRMS), Amman, Jordan

**Keywords:** Behçet's disease (BD), Vasculitis, Pseudoaneurysms (PSA), Open surgical repair

## Abstract

**Introduction and importance:**

Behçet's Disease is a chronic, multi-systemic vasculitis of unknown aetiology that classically presents with a triple-symptom complex of recurrent oral ulcers (aphthous stomatitis), genital ulcers and uveitis (chronic iridocyclitis)**.** Vascular involvements of Behçet disease include arterial and venous thrombosis, formation of an unusual aneurysm and arterial occlusion, known as vasculo-Behçet's disease**.**

**Case presentation:**

A 21-year-old male presented with recurrent painful oral ulcers and bilateral lower limb deep vein thrombosis. Also, he presented with thigh pain and swelling, diagnosed as a giant pseudoaneurysm of the right SFA. CT angiography revealed an 80.2 × 76.9 × 69 mm pseudoaneurysm. He was scheduled to undergo emergency surgery due to severe, intractable pain. The pseudoaneurysm was excluded, and using a reversed basilic vein graft interposition, we performed a femoral-femoral bypass from the proximal femoral artery to a distal superficial femoral artery. Postoperatively, the patient had an uneventful course; pain and swelling subsided.

**Clinical discussion:**

The diagnosis of Behçet's disease is based on clinical criteria consisting of combinations of symptoms due to the lack of universally recognised pathognomonic laboratory tests. Arterial complications of Behçet's disease occur in 1 % to 7 % of patients, with a male predominance. Immunosuppressants, such as cyclophosphamide or azathioprine, represent the mainstay treatment of Behçet's disease and should always be considered to achieve complete remission, prevent recurrences, and reduce the risk of postoperative complications**.**

**Conclusion:**

Pseudoaneurysm is the most common presentation of arterial complications of Vasculo- Behçet's disease and should be kept in mind to prevent significant morbidity and mortality.

## Highlights

• Behçet's Disease (BP) is a chronic, inflammatory multi-systemic vasculitis of unknown aetiology.
• BD has a broad clinical spectrum of mucocutaneous, ocular, articular, neurologic, gastrointestinal and vascular involvement.
• Vasculo-Behçet's disease includes arterial and venous thrombosis, formation of an unusual aneurysm and arterial occlusion.
• Periodic follow-up and appropriate treatment of Behcet's disease is mandatory; to prevent irreversible organ damage.
• Arterial pseudoaneurysm is a life-threatening manifestation of vasculo-Behçet's disease.

## 1 Introduction and importance

Vasculitis is a nonspecific term for a large and heterogeneous group of rare disorders characterised by acute and chronic inflammatory changes in the vessel wall. There are numerous disorders; in most, aetiology remains unknown.

Behçet's disease (BP) is a chronic, inflammatory multi-systemic vasculitis of unknown aetiology that classically presents with a triple-symptom complex of recurrent oral ulcers (aphthous stomatitis), genital ulcers and uveitis (chronic iridocyclitis) [1]. Behçet's disease follows a chronic course with unpredictable inflammatory attacks and periods of remission. However, relapses and remissions are characteristic [2].

Several other manifestations, including articular, neurological, gastrointestinal, and vascular, have been described. Articular involvement is manifested with non-deforming arthritis and is observed in approximately half of the patients [3]. Neurological manifestation is one of the most serious complications of Behçet disease due to its severe prognosis [4]. Gastrointestinal involvement is manifested by punched-out mucosal ulcers that occur predominantly in the ileocecal region but can occur throughout the gastrointestinal tract [5].

Clinically, vascular involvements of Behçet disease include arterial and venous thrombosis, formation of an unusual aneurysm, particularly those affecting the pulmonary arteries and arterial occlusion, known as vasculo-Behçet's disease (v-BD) [6].

An arterial aneurysm is a localised enlargement of an artery >50 % of its original diameter. Aneurysms can be either true or false. A true aneurysm is a localised enlargement of an artery, including all three arterial wall layers (intima, media and adventitia). On the other hand, an pseudoaneurysm (PSA), also known as a false aneurysm or out-pouching of the vessel, is a potentially lethal vascular lesion caused by disruption of the arterial wall (intimal and medial layers). Therefore, a pseudoaneurysm is a sac containing turbulent blood flow connected to the artery's lumen through a defect in its wall. It is usually surrounded by haematoma without an epithelial wall layer; a fibrin/platelet cross-links wall eventually forms. True aneurysms are divided into two types fusiform and saccular. The fusiform type is wide in the middle and tapers at both ends, classically an abdominal aortic aneurysm (AAA). The saccular type may be almost spherical and projects from one point on the arterial wall but still contains all three wall layers. Finally, a giant pseudoaneurysm is a vascular lesion >5 cm in diameter [7].

The risk factors for an arterial aneurysm include smoking, male sex, hypertension, hypercholesterolaemia, coronary heart disease and peripheral artery disease. Other risk factors include vasculitis and connective tissue disease (e.g., vasculo-Behçet's Disease or Marfan's syndrome). Clinically, the presentation of an arterial aneurysm varies from an asymptomatic mass detected incidentally on routine physical examination to acute limb-threatening ischemia. In addition, patients may notice a pulsatile swelling or symptoms such as pain over the swelling from compression of other surrounding structures [7,14].

We report our experience at the Department of Vascular Surgery with a 21-year-old male with active vasculo-Behçet's disease presented with a giant pseudoaneurysm of the right superficial femoral artery, which was treated with pseudoaneurysm exclusion. Using a reversed basilic vein graft interposition, we performed a femoral-femoral bypass from the proximal femoral artery to a distal superficial femoral artery. The patient gave his consent to publish his case report. This case report has been reported in line with the SCARE 2020 Criteria [8].

## 2 Case presentation

A 21-year-old male presented to the emergency department with a three-day right-sided inner thigh pain and swelling history. The patient had no history of trauma or local surgery. He was known to have recurrent painful oral ulcers. Eighteen months before presentation, he developed bilateral lower extremity deep vein thrombosis and was initially treated with low molecular weight heparin (LMWH), which overlapped with warfarin. He denied skin rashes, fevers, chills, malaise, weight loss, poor appetite, red eyes, chest pain, shortness of breath, changes in urination, nausea/vomiting/diarrhoea, Reynaud's phenomenon, joint swelling or photosensitivity. He had a positive family history of rheumatoid arthritis in the mother and Hodgkin lymphoma in the father. He currently smokes 15 cigarettes a day and is not married. No allergy to any drug has been reported. The patient had never undergone any surgical intervention.

On examination, he was afebrile. His vital signs on arrival were: heart rate 78/min, respiratory rate 16/min, blood pressure 110/70 mmHg, and O2 saturation on room air was 100 %.

**Fig. 1:**
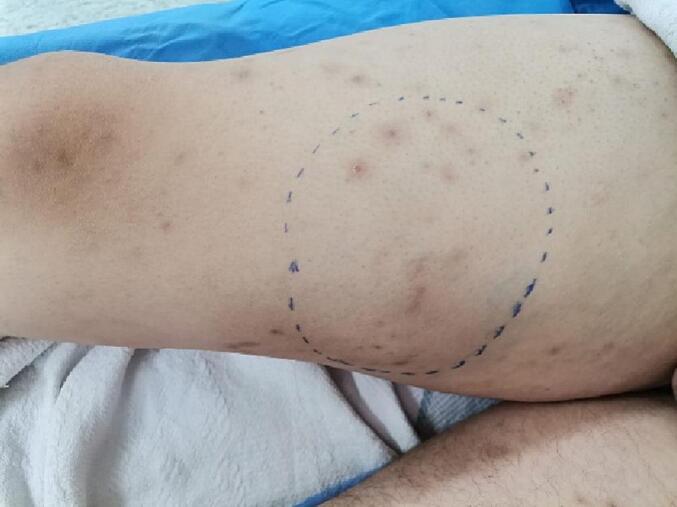
A well-defined, pulsatile mass in the right medial thigh measuring 8.0 × 7.0 cm. Numerous reddish-purple nodular scars on both lower extremities were seen. (For interpretation of the references to color in this figure legend, the reader is referred to the web version of this article.)

Clinical examination revealed a well-defined, pulsatile swelling in the right medial thigh without palpable thrill or audible bruit. The lesion was 8.0 × 7.0 cm, soft and tender [Fig. 1]. Right popliteal, posterior tibial and dorsalis pedis pulses were all impalpable.

There were numerous reddish-purple nodular scars on both lower extremities. The oral mucosa was moist, and no ulcerations were observed. Head and neck examination revealed left submandibular and cervical lymphadenopathy. Respiratory, abdominal, and neurological examinations were unremarkable.

**Fig. 2:**
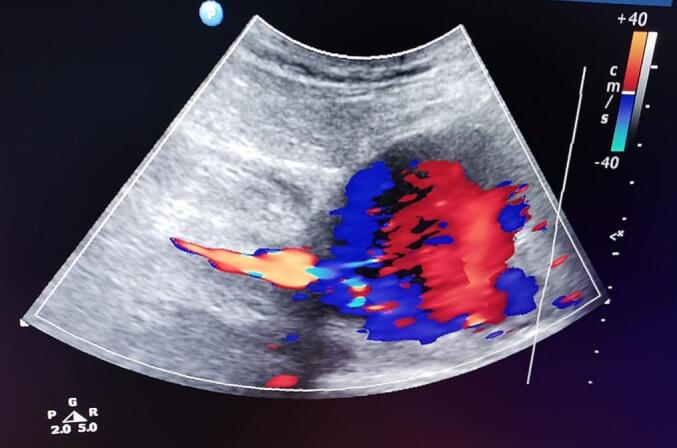
A Color Doppler Ultrasound showed about 80 × 75 × 70 mm pseudoaneurysm at the mid-level of the right SFA with a wide neck leading to compression of the middle segment of the SFA.

Arterial and Venous Doppler Ultrasound showed non-occlusive deep vein thrombosis (DVT) in both common femoral veins with good filling, patent right common femoral artery (CFA) and patent proximal right superficial femoral artery (SFA) with triphasic signals. In addition, about 80 × 75 × 70 mm pseudoaneurysm is noted at the mid-level of the right SFA with a wide neck leading to compression of the middle segment of the SFA. However, the distal right SFA was patent with a biphasic signal. In addition, distal vessels, anterior and posterior tibial arteries, were patent with biphasic signals [Fig. 2].

Laboratory studies revealed a severe inflammatory reaction; WBC was 19,000 /μL; the erythrocyte sedimentation rate (ESR) was 95 mm/1st h (normal range < 25), and the C-reactive protein (CRP) was 44 mg/L (normal range 0.0–5.0). A complete blood count (CBC) revealed normal hematocrit and platelet count. The coagulation profile: Prothrombin time (PT) was 16.5 s; Activated Partial Thromboplastin Clotting Time (aPTT) was 36.5 s; and an international normalised ratio (INR) was 1.7. The HLA-B51 was negative. Anticardiolipin (aCL) IgG and IgM antibodies were negative. The pathergy test was positive. The ophthalmic assessment was performed without significant pathological findings. Fine-needle aspiration cytology of cervical lymphadenopathy was negative for malignancy. The patient fulfilled the International Criteria for Behçet's Disease (ICBD) by rheumatologic assessment.

**Fig. 3:**
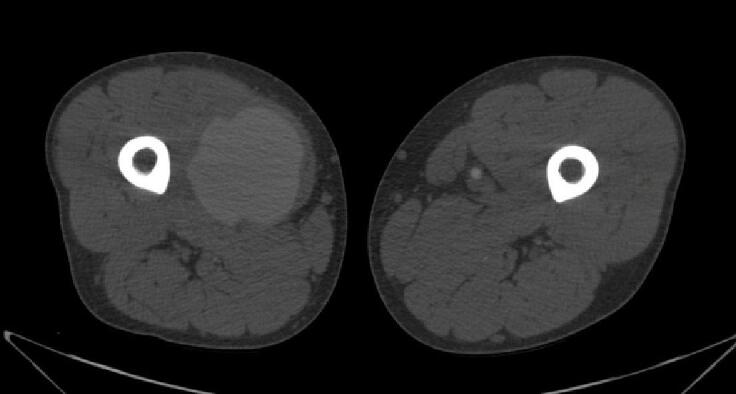
A Computed Tomographic Angiography (CTA) of the Lower Extremities: Axial view demonstrating a giant pseudoaneurysm of the right superficial femoral artery (SFA).

**Fig. 4:**
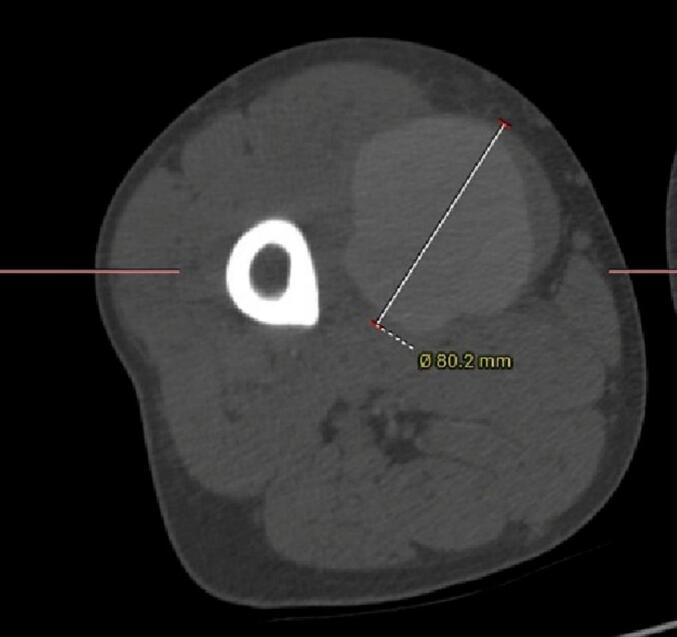
A CT angiography of right the lower limb: Axial view demonstrating a giant pseudoaneurysm of the right SFA with a maximal diameter of 80.2 mm.

**Fig. 5:**
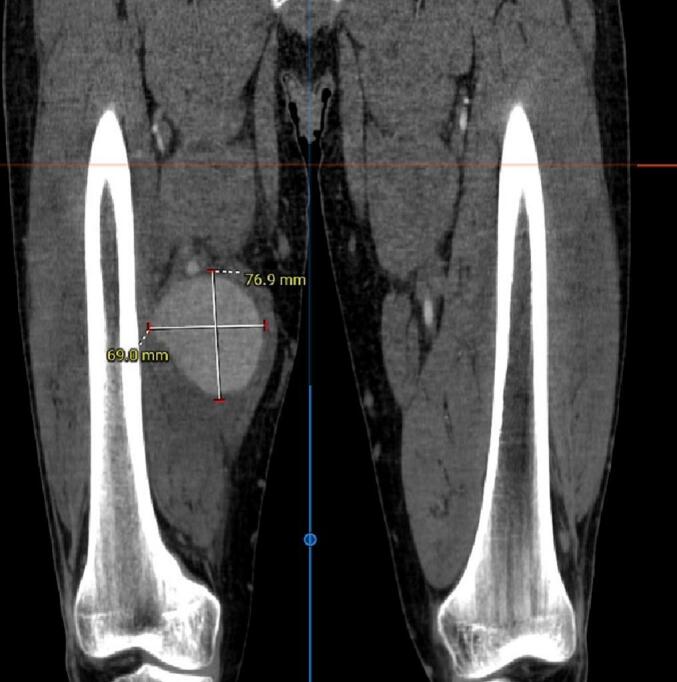
A CT angiography of the lower extremities: Coronal view demonstrating a giant pseudoaneurysm of the right superficial femoral artery (SFA) with a diameter of 76.9 × 69.0 mm.

**Fig. 6:**
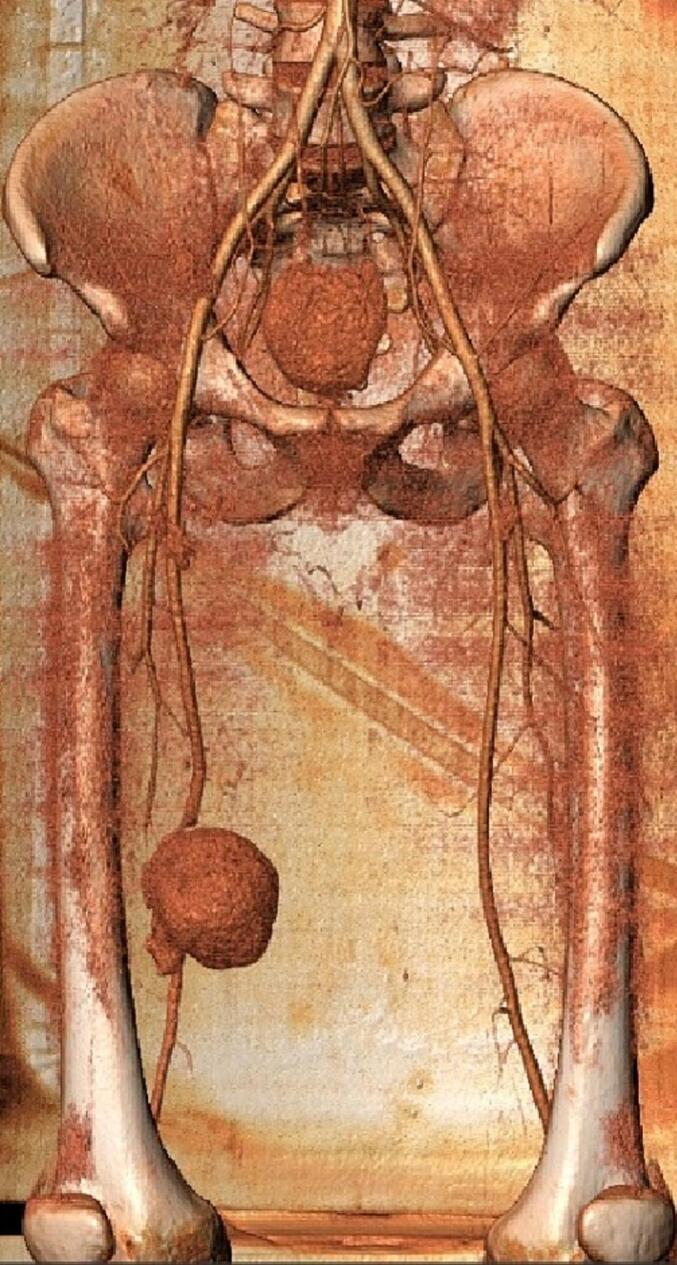
A CT angiography with 3D reconstruction of the pelvic and lower extremities demonstrating a giant pseudoaneurysm of the SFA.

Based on clinical data and investigation results, the diagnosis of Vasculo-Behçet's Disease was established, and the patient was started on immunosuppressive therapy for his active disease. The decision was then made to arrange emergency surgery due to severe, intractable pain and a high risk of pseudoaneurysm rupture and lower limb ischemia. In preparation for surgery, a chest, abdomen and lower extremities computed tomography angiography (CTA) was performed to exclude aneurysmal lesions. CTA revealed an 80.2 × 76.9 × 69 mm pseudoaneurysm in the right superficial femoral artery [Fig. 3, Fig. 4, Fig. 5, Fig. 6].

Preoperatively, the patient received a pulse of methylprednisolone 1 g/day for 3 days and cyclophosphamide 500 mg intravenously.

After receiving confirmation of consent from the patient, the preoperative assessment included an imaging review and planning of proximal and distal anastomosis points. In addition, a venous examination of the right upper limb was also done to select a suitable autologous vein for use as a graft for arterial reconstruction. Preoperative Doppler ultrasound revealed a patent right basilic vein with good calibre.

**Fig. 7:**
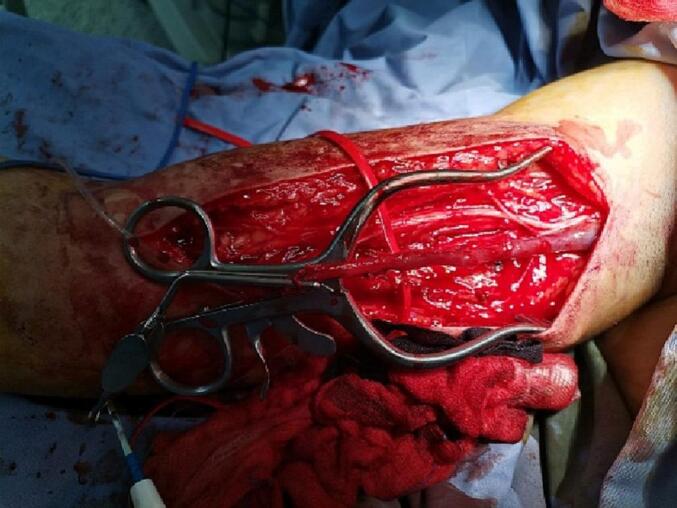
An intraoperative photograph showing the right basilic vein for use in the reverse technique to femoral artery repair after pseudoaneurysm was excluded.

**Fig. 8:**
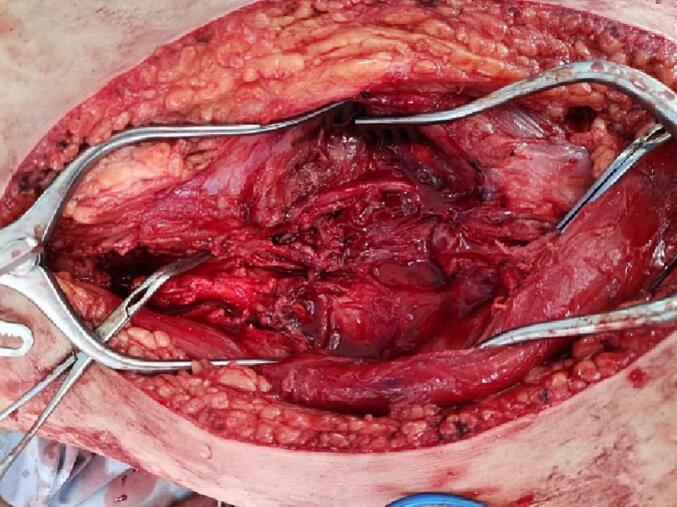
An intraoperative photograph showing a giant pseudoaneurysm exclusion with control of the proximal and distal segments of the right superficial femoral artery (SFA).

**Fig. 9:**
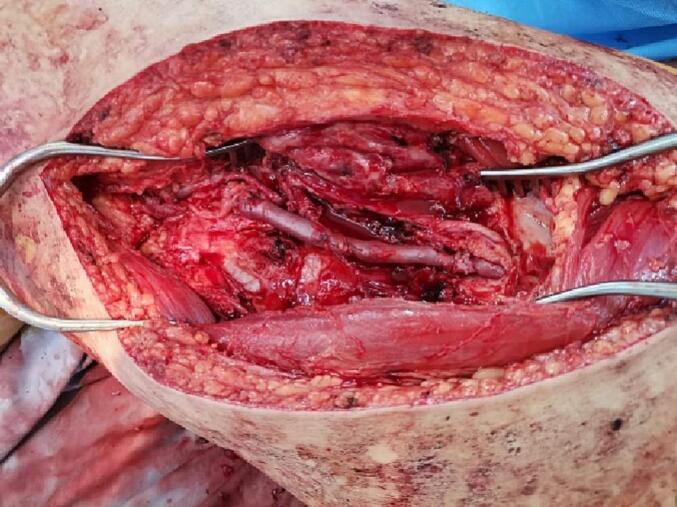
An intraoperative photograph showing femoral-femoral bypass using an interposition of reversed basilic vein graft.

General anaesthesia was performed by endotracheal intubation. Prophylactic antibiotics were administered. A urinary catheter had already been inserted. The patient was positioned supine with the right knee rested on a sterile saline bag to allow slight hip and knee joint flexion. The right upper and lower extremities were prepped and draped routinely. A medial approach was used to expose the right superficial femoral artery, in which a giant pseudoaneurysm was identified. A longitudinal mid-thigh incision was made with a subsequent opening layer opening to reveal the adductor muscles anteriorly and the sartorius muscle posteriorly. Proximal and distal control of the right SFA were performed. An incision along the pre-marked right basilic vein was made, followed by harvesting the vein to be used in reversed technique to repair the femoral artery after excluding a pseudoaneurysm. [Fig. 7, Fig. 8]. The patient underwent open surgical exclusion of a pseudoaneurysm. Then, using a reversed basilic vein graft interposition, we performed a femoral-femoral bypass from the proximal femoral artery to a distal superficial femoral artery [Fig. 9].

Postoperatively, the patient had an uneventful recovery; pain and swelling subsided immediately. During his hospitalisation, the patient received oral antimicrobial treatment and oral calcium plus vitamin D. Oral prednisolone and intravenous cyclophosphamide were continued postoperatively and adjusted according to the disease activity and rheumatologic assessment.

The patient was discharged from the hospital on the 5th postoperative day with palpable peripheral pulses and triphasic arterial Doppler waveform of the lower limb. Pre-discharge instructions were given for strict smoking cessation advice and regular follow-up in the clinic.

He was placed on warfarin (Coumadin®) 5 mg daily, clopidogrel (Plavix®) 75 mg daily, atorvastatin (Lipitor®) 10 mg at bedtime, and prednisolone (Deltasone®) 1 mg/kg/day (Initial daily dose 60 mg/day); begin tapering dose after 2 weeks; with slow tapering over 6 weeks.

After two weeks, the patient returned to the clinic in good condition, without complications. At one-month and three-month follow-up visits, the patient had no symptoms or physical signs of aneurysm recurrence associated with normal ESR and CRP values. In addition, arterial Doppler ultrasound performed at 3 months and computed tomography angiography (CTA) performed at 6 months after the procedure confirmed pseudoaneurysm exclusion, the vein bypass graft patency, and the absence of recurrences.

## 3 Clinical discussion

Behçet's disease (BD) was described in 1937 by the Turkish dermatologist Hulusi Behçet as a multisystem vasculitis classically manifested by oral and genital aphthosis and uveitis [9]. BD is most prevalent along the historic Silk Road trade route in Turkey, the Mediterranean basin, and the Middle and Far East. The disease predominates in young men between 20 and 40 years [10].

The diagnosis of Behçet's disease is based on clinical criteria consisting of combinations of symptoms due to the lack of universally recognised pathognomonic laboratory tests. In 2014, experts from 27 countries conducted a collaborative study to determine a standard diagnostic measure of Behçet's Disease. As a result, a new set of International Criteria for Behçet's Disease (ICBD) was proposed to diagnose and classify BD [see Table 1] [11].

**Table 1:** The Revised International Criteria for Behçet's Disease Scoring System.

Sign/symptom	Points
Ocular lesions	2
Genital aphthosis	2
Oral aphthosis	2
Skin lesions	1
Neurological manifestations	1
Vascular manifestations	1
Positive pathergy test	1

The pathergy test is optional. However, where a pathergy test is conducted, 1 extra point may be added for a positive result.

A score of ≥ 4 indicated a definitive diagnosis of BD.

Vasculo-Behçet's disease (v-BD) has different clinical presentations, such as venous thrombosis, arterial pseudoaneurysm, aneurysm formation, and occlusive arterial disease. The venous system is the most commonly affected site, and superficial or deep vein thrombosis is the most frequent vascular issue [12]. Arterial involvements are less common than venous complications, occurring in about 1 % to 7 % of patients. Nevertheless, vascular involvement is a major cause of morbidity and mortality in Behçet's disease [13,14].

The most common arterial presentation in Behçet's disease patients is pseudoaneurysm development, with a significantly higher frequency than true aneurysm formation. Behçet's aneurysms tend to be multiple and can involve any artery. The initial pathology in the affected artery is active arteritis, mainly around the perivascular (vasa vasorum), which may lead to transmural necrosis, progressive thickening of the vessel wall, aneurysmal dilatation, and eventually, perforation of the vessel wall and formation of pseudoaneurysm. In patients with venous involvement of Behçet's disease, DVT is thought to be due to inflammation of the vessel wall rather than hypercoagulability [13,14].

Inflammation of the blood vessels and endothelial dysfunction play a role in the pathogenesis of vasculitis. Involvement of the large vessels is observed in up to 40 % of Behçet's patients. Arterial pseudoaneurysms have also been associated with high levels of CRP and ESR [15]. Patients with Behçet's disease, especially men, should be screened for abdominal aortic aneurysms during exacerbations [16].

Under the auspices of the Standing Committee on Clinical Affairs of the European League Against Rheumatism (EULAR), different new treatment modalities have been studied in patients with Behçet's disease by experts from various disciplines, including internal medicine, rheumatology, ophthalmology, neurology, gastroenterology, dermatology and vascular surgery. As a result, recommendations regarding the medical and surgical intervention for vascular complications of Behçet's disease have been modified [17].

In patients with Vasculo-Behçet's disease, immunosuppressants, such as cyclophosphamide or azathioprine, represent the mainstay treatment. Immunosuppressive therapy should always be considered to achieve complete remission, prevent recurrences, and reduce the risk of postoperative complications [18]. Saadoun et al. studied the long-term outcome of 101 patients with arterial abnormalities among a cohort of 820 (12.3 %) Behçet's disease patients. They concluded that postoperative complications were significantly less frequent in patients receiving immunosuppressants, improving the prognosis [19].

One of the most controversial issues regarding managing Behçet's disease is whether arterial and venous thrombosis should be treated with immunosuppressant therapy, anticoagulants, or both [18].

Anticoagulants may be added to prevent relapses of venous thrombosis, post-thrombotic syndrome and recurrent arterial occlusive events, provided that the overall risk of bleeding is low and pulmonary artery aneurysms are excluded. (Level of evidence: III; strength of recommendation: C) [17].

Surgery or endovascular stenting should not be delayed if the patient is symptomatic. (Level of evidence: III; strength of recommendation: C) [17].

Peripheral artery aneurysms require emergency surgery or endovascular stenting unless they are small, asymptomatic and carry a low risk of rupture [18].

Medical treatment with high-dose corticosteroids and cyclophosphamide may be appropriate for such small aneurysms. Ideally, medical treatment should start before an aneurysm repair is attempted [18].

In conclusion, the EULAR (2018 update) Recommendations for the management of Behçet's disease (BD) developed five overarching principles [17]:

(1) BD is a multi-systemic vasculitis of unknown aetiology that classically follows a relapsing and remitting course. Therefore, treatment should promptly inhibit inflammatory exacerbations and recurrences from preventing irreversible organ damage. As a result, immunosuppressive therapy is usually necessary to achieve this effect.
(2) A multidisciplinary approach is essential for optimal care.
(3) Treatment should be individualised according to age, type and severity of organ involvement, and patient preferences.
(4) Vascular, neurological, ocular and gastrointestinal manifestations may be associated with a poor prognosis.
(5) The manifestation of BD may subside and ameliorate over time in many patients. Therefore, treatment can be tapered and even stopped during the course of the disease.

Therapeutic modalities for pseudoaneurysms require considering multiple factors, including aetiology, size, location, accessibility, and availability of vascular intervention facilities. Therefore, depending on the case, conservative management by ultrasound-guided compression and thrombin injection, endovascular stent and surgery are the different approaches for management [20].

Endovascular stents have the advantage of reducing the incidence of postoperative complications by limiting endothelial injury to the already fragile arterial wall. However, there are anatomical limitations, and aneurysm formation most commonly occurs at arterial puncture sites or along the edge of a stent graft. Covered-stent grafts have recently been used to exclude pseudoaneurysms in some selected cases, preserving patients from mortality and morbidity due to surgery. But They may be complicated by stent fracture and migration [21].

The choice between open surgical and endovascular interventions can be made according to the size and location of the pseudoaneurysm and the surgeon's experience. However, open surgical options should be emphasised as the first-line option in patients with vasculo-Behçet's disease (v-BD) unless the cardiopulmonary risk is prohibitive [20].

The long-term consequences of open surgical revascularization and reconstruction of the vascular complications of Behçet's disease have yet to be clearly defined. Because of this lack of follow-up data or patients with vasculo-Behçet's disease (v-BD) who underwent arterial reconstruction, there has been some debate as to whether autologous vein graft reconstruction or prosthetic graft reconstruction represents the optimal treatment modality for patients whose arterial repair requires a vascular conduit. Autologous vein grafts provide excellent short- and long-term patency, durability, graft resistance to infection, and limb salvage. However, many authors prefer prosthetic grafts because autologous vein grafts have a higher risk of thrombosis in Behçet's patients [20].

Antithrombotic therapy in patients with peripheral arterial diseases (PAD) includes antiplatelet agents and anticoagulation therapy. Antiplatelet agents are used for secondary prevention of limb-related and general cardiovascular events. According to the recommendations of the European Society of Cardiology (ESC) and the European Society for Vascular Surgery (ESVS) for the diagnosis and treatment of peripheral arterial disease, antithrombotic therapy includes [22]:

1) Long-term single antiplatelet therapy (SAPT) is recommended in all patients who have undergone revascularization. (Level of evidence: I; strength of recommendation: C).
2) Single antiplatelet therapy is recommended after infra-inguinal bypass surgery. (Level of evidence: I; strength of recommendation: A).
3) In patients requiring antiplatelet therapy, clopidogrel may be preferred over aspirin. (Level of evidence: II-b; strength of recommendation: B).
4) Vitamin K antagonists may be considered after autologous vein infra-inguinal bypass. (Level of evidence: II-b; strength of recommendation: B).

In this case, a young patient presented with a giant pseudoaneurysm, so open surgical exclusion and repair were attempted considering available resources. We preferred autologous vein graft reconstruction instead of synthetic materials to avoid the risk of graft infection and occlusion. Surgical prosthetic infection is a major cause of morbidity and mortality.

Anastomotic pseudoaneurysm is a late complication of prosthetic graft reconstruction for patients with vasculo-Behçet's disease (v-BD) who underwent previous successful arterial revascularization and is the most common serious complication [23].

## 4 Conclusion

Periodic follow-up and appropriate treatment of Behçet's disease (BD) is mandatory; to prevent irreversible organ damage, particularly in the early, active stages of Behçet's disease. One of the devastating and life-threatening manifestations of Behçet's disease (BD) is its vascular complications. Pseudoaneurysms (PSA) are the most common presentation of arterial complications of Behçet's disease and should be kept in mind to prevent significant morbidity and mortality from bleeding and limb ischemia. Treatment of vasculo-Behçet's disease (v-BD) manifestations varies with the affected area and the specific event type (venous versus arterial involvement). Treatment is mainly based on suppressing the exacerbation and recurrence of inflammatory attacks. Anti-TNF agents (mainly infliximab) may be considered for refractory cases and represent a life-saving treatment. For arterial pseudoaneurysms, pharmacological therapy is based primarily on immunosuppressants (cyclophosphamide or azathioprine), often combined with corticosteroids and surgery. Surgery or endovascular stenting should not be delayed if the patient is symptomatic.

## Source of funding

This study has no sponsors and is self-funded.

## Ethical approval

Ethical approval has been taken from The Ethical Committee at King Hussein Medical Center, Jordanian Royal Medical Services (JRMS), Amman, Jordan.

The reference number is 4/2023–49.

The date of approval by the ethics committee 23/5/2023.

## Consent

Written informed consent was obtained from the patient for publication of this case report and accompanying images. A copy of the written consent is available for review by the Editor-in-Chief of this journal on request.

## CRediT authorship contribution statement

Interpretation of the CT images:

• Mohammad A. Al-Doud.

Pre-Operative care:

• Anas N. Al-Nusairat, Nael M. Al-shanableh. Moath M. El-Sageer.

Operation:

• Main Surgeon: Mohammad A. Al-doud.

Assistants Surgeon:

• Anas N. Al-Nusairat, Nael M. Al-shanableh, Omar I. Thabcem.

Post-Operative Care:

• Anas N. Al-Nusairat, Nael M. Al-shanableh, Sadeq M. Da'meh.

Drafting the manuscript:

• Mohammad A. Al-doud, Anas N. Al-Nusairat.

Revising the manuscript critically for important intellectual content:

• Mohammad A. Al-doud, Nael M. Al-shanableh, Omar I. Thabcem.

Approval of the version of the manuscript to be published (the names of all authors must be listed):

• Mohammad A. Al-doud, Anas N. Al-Nusairat, Nael M. Al-shanableh, Sadeq M. Da'meh, Omar I. Thabcem, Moath M. El-Sageer,

## Registration of research studies

The authors don't need to register this work.

## Guarantor

Dr. Mohammad A. Al-Doud.

## Declaration of competing interest

The authors declare that they have no known competing financial interests or personal relationships that could have appeared to influence the work reported in this paper.
